# Prognostic prediction of head and neck squamous cell carcinoma: Construction of cuproptosis‐related long non‐coding RNA signature

**DOI:** 10.1002/jcla.24723

**Published:** 2022-10-03

**Authors:** Qi Huang, Quanjie You, Ning Zhu, Zhenhua Wu, Zhenfei Xiang, Kaiyuan Wu, Jianjun Ren, Yihua Gui

**Affiliations:** ^1^ Department of Otorhinolaryngology Head and Neck Surgery Ningbo Medical Center Lihuili Hospital Ningbo China; ^2^ Department of Otorhinolaryngology Head and Neck Surgery Lihuili Hospital affiliated to Ningbo University Ningbo China; ^3^ Department of Otolaryngology Second Hospital of Ninghai County Ningbo China; ^4^ Department of Radiation Oncology Lihuili Hospital affiliated to Ningbo University Ningbo China

**Keywords:** cuproptosis, head and neck squamous cell carcinoma, long noncoding RNA, prognostic

## Abstract

**Background:**

Recently, a new type of programmed cell death, cuproptosis, has been identified to play important role in the progression of tumors. We constructed a cuproptosis‐related long non‐coding RNA (lncRNA) signature to predict the prognostic significance for head and neck squamous cell carcinoma (HNSCC).

**Methods:**

The risk model was developed based on differentially expressed lncRNAs associated with cuproptosis. Principal component analysis was used to assess the validity. The Kaplan–Meier curves were analyzed to compare the overall survival (OS), disease‐specific survival (DSS), and progression‐free survival (PFS) values. The multivariate and univariate Cox regression analyses were used to evaluate the prognostic efficiency. Furthermore, the functional enrichment, immune cell infiltration, tumor mutation burden (TMB), and sensitivity toward chemotherapy were also explored.

**Results:**

Six cuproptosis‐related lncRNAs (AL109936.2, CDKN2A‐DT, AC090587.1, KLF3‐AS1, AL133395.1, and LINC01063) were identified to construct the independent prognostic predictor for HNSCC. The area under the curve and C‐index values obtained using the risk model were higher than the values corresponding to the clinical factors. Analysis of Kaplan–Meier curves indicated that the OS, PFS, and DSS time recorded for the patients in the low‐risk group were higher than the corresponding values recorded for the patients belonging to the high‐risk group. By functional enrichment analysis, we observed that differentially expressed genes were enriched in the immune response and tumor‐associated pathways. The patients characterized by a low‐risk score exhibited better immune cell infiltration than the patients belonging to the other group. We also observed that the sensitivity of the individuals belonging to the low‐risk group to chemotherapeutic agents (cisplatin, docetaxel, and paclitaxel) was higher than the sensitivity of those in the other group.

**Conclusions:**

A cuproptosis‐related lncRNA‐based signature that functioned as an independent prognosis predictor for HNSCC patients was constructed. The chemosensitivity of individual patients can be potentially predicted using this signature.

## Graphical abstract

In this study, HNSCC clinical data and cuproptosis‐associated genes data were extracted from the public database. Prognostic differential genes were screened, and six cuproptosis‐associated LncRNAs signature was constructed. The developed signature was characterized by an independent prognostic value for the case of the total HNSCC cohort. The survival duration of the patients suffering from HNSCC and characterized by a high risk score was shorter than the survival time recorded for the patients characterized by low‐risk scores. The prognosis for the low‐risk score samples was better than the prognosis for the high‐risk score samples belonging to the stage III + IV subgroups. Enrichment of several immune‐related biological processes was observed in cuproptosis‐related lncRNAs by GO and KEGG analysis; The checkpoint, human leukocyte antigen (HLA), T cell co‐stimulation, inflammation‐promoting, and type II interferon response pathways were enriched in the low‐risk group. The low‐TMB group patients were more likely to survive for a longer period of time than those in the high‐TMB group. The high‐risk patients were more sensitive to cisplatin‐, docetaxel‐, and paclitaxel‐based treatment schemes.

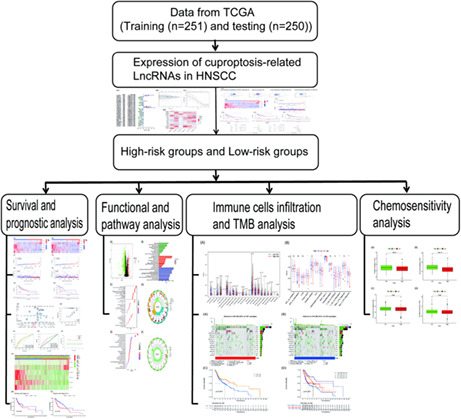

## 1 INTRODUCTION

Head and neck squamous cell carcinoma (HNSCC) is characterized by the presence of a multi‐source group of malignant tumors that have their origin in the squamous epithelium cells of the larynx, pharynx, and oral cavity. HNSCC is the sixth leading malignancy worldwide, and 600,000 new cases are reported each year (accounting for 5.5% of the total systemic cancer cases).^1^, ^2^ The origin of most HNSCC cases can be traced back to viral infections caused by high‐risk oncogenic human papillomavirus (HPV), genetic inheritance, tobacco‐derived carcinogens, and excessive alcohol consumption. The primary treatment options, including chemotherapy, surgery, and radiotherapy, depend on the clinical stage and conditions of individual HNSCC patients.^3^ Although great progress in the fields of immunotherapy and targeted therapy has been made in recent years, the rate of 5‐year survival remains low (<50%), and this can be attributed to the difficulty in determining the location of tumors and the absence of distinct early‐stage clinical symptoms. Hence, in most cases, patients are diagnosed when they are at an advanced stage of HNSCC.^4^ Thus, it is important to identify the molecular mechanisms associated with the onset and progression of HNSCC and identify novel prognostic risk models that can be used for the effective and reliable management of this type of cancer.

Long non‐coding RNAs (lncRNAs) contain approximately 200–100,000 nucleotides.^5^ Although lncRNAs do not encode proteins or peptides, they have attracted widespread attention as they regulate various biologically important processes, such as activation of cells, the proliferation of cells, metabolism, development of immunity, and cell death.^6^, ^7^ Numerous researchers have reported that lncRNAs are potential therapeutic targets and biomarkers that can be used for the diagnosis and treatment of cancer.^8^, ^9^ Numerous dysregulated lncRNAs dictate the processes associated with the onset and advancement of HNSCC.^10^, ^11^ For example, lncMX1‐215 negatively regulates the process of immunosuppression by hindering the process of H3K27 acetylation.^12^ LTSCCAT promotes the metastasis of tongue squamous cell carcinoma by targeting the miR‐103a‐2‐5p/SMYD3/TWIST1 axis.^13^ Wang et al.^14^ reported that MIR31HG targeted HIF1A and P21 to facilitate the proliferation of the HNSCC cells and the process of tumorigenesis by regulating the cell cycle. The observations reveal that lncRNAs can be used as potential diagnostic biomarkers to develop targeted therapeutic methods. They can also play prognostic roles in the case of HNSCC. Novel therapeutic targets can be identified, and effective drugs for HNSCC patients can be developed by conducting further studies on the prognostic roles of lncRNAs.

A new mechanism resulting in programmed cell death was reported by Tsvetkov et al.^15^ in 2022. The pathway was known as cuproptosis, and it was different from apoptosis, autophagy, necroptosis, pyroptosis, and ferroptosis. Under conditions of cuproptosis, copper combines with the lipoylated components of the tricarboxylic acid (TCA) cycle. The formation of lipoylated protein aggregates and the loss of iron–sulfur cluster proteins are observed under these conditions. Cuproptosis proceeds under conditions of toxic protein stress, resulting in cell death. Copper is a trace element necessary for numerous biological processes and the normal functioning of various metabolic enzymes.^16^ It is a double‐edged sword that affects the vital processes occurring in biological systems. The redox properties of copper promote various physiological processes, and in some cases, copper functions as a toxic substance.^17^ Copper promotes the growth and metastasis of tumors, and an imbalance in copper homeostasis results in the onset and progression of cancer.^18^, ^19^ The copper levels in the serum of tumor patients are significantly different from the copper levels recorded in the serum of healthy people.^19^ To date, studies on the mechanism of cuproptosis associated with HNSCC have not been conducted, and the influence of cuproptosis‐related lncRNAs on the onset and progression of HNSCC is unclear. Several genes related to cuproptosis have been identified, and the analysis of the properties and functions of these genes can help in prognosis prediction.

To explore effective prognostic model and reveal the association of cuproptosis in HNSCC, we identified the differentially expressed cuproptosis‐related lncRNAs and used six cuproptosis‐related lncRNAs to construct the risk signature. The developed model could be used to improve the reliability of prognostic risk stratification and develop therapeutic strategies. We analyzed the mechanism following which the cuproptosis‐related lncRNAs exerted their effects on the onset and advancement of HNSCC by using the functional enrichment analysis. We also studied the relationship between the risk score and clinicopathologic features, sensitivity toward chemotherapy, immune cell infiltration levels, and tumor mutation burden (TMB).

## 2 MATERIALS AND METHODS

### 2.1 Collection of data

We downloaded the transcriptome profiles (fragments per kilobase million values) of 501 HNSCC tissue samples (database: The Cancer Genome Atlas (TCGA), July 1, 2022). The database was accessed at https://portal.gdc.cancer.gov/. The relevant prognostic data and clinicopathological information (including data on survival status, age, N stage, histologic grade, lymph node metastasis, gender, T stage, clinical stage, overall survival (OS), disease‐specific survival (DSS), and progression‐free survival (PFS) values) were also extracted (Table 1). Five hundred and one patients were classified into two cohorts (training (*n* = 251) and testing (*n* = 250) (ratio: 1:1)) for the preliminary evaluation of the process associated with signature identification. The mode of classification was random.

**TABLE 1:** Clinical characteristics of the patients with HNSCC in the training and testing cohort

Covariates	Type	Total	Train	Test	*p* Value
Age	≤ 60	246 (49.1%)	114 (45.6%)	132 (52.59%)	0.1401
	> 60	255 (50.9%)	136 (54.4%)	119 (47.41%)	
Gender	Female	133 (26.55%)	71 (28.4%)	62 (24.7%)	0.403
	Male	368 (73.45%)	179 (71.6%)	189 (75.3%)	
Histologic grade	G1	61 (12.18%)	32 (12.8%)	29 (11.55%)	0.9162
	G2	299 (59.68%)	149 (59.6%)	150 (59.76%)	
	G3	119 (23.75%)	60 (24%)	59 (23.51%)	
	G4	2 (0.4%)	1 (0.4%)	1 (0.4%)	
	Unknown	20 (3.99%)	8 (3.2%)	12 (4.78%)	
T	T1	33 (6.59%)	16 (6.4%)	17 (6.77%)	0.2799
	T2	144 (28.74%)	65 (26%)	79 (31.47%)	
	T3	130 (25.95%)	65 (26%)	65 (25.9%)	
	T4	179 (35.73%)	99 (39.6%)	80 (31.87%)	
	Unknown	15 (2.99%)	5 (2%)	10 (3.98%)	
M	M+	25 (4.99%)	10 (4%)	15 (5.98%)	0.535
	M0	471 (94.01%)	238 (95.2%)	233 (92.83%)	
	Unknown	5 (1%)	2 (0.8%)	3 (1.2%)	
N	N0	239 (47.7%)	128 (51.2%)	111 (44.22%)	0.1569
	N1	80 (15.97%)	39 (15.6%)	41 (16.33%)	
	N2	153 (30.54%)	74 (29.6%)	79 (31.47%)	
	N3	7 (1.4%)	1 (0.4%)	6 (2.39%)	
	Unknown	22 (4.39%)	8 (3.2%)	14 (5.58%)	
Stage	I	19 (3.79%)	12 (4.8%)	7 (2.79%)	0.3349
	II	95 (18.96%)	44 (17.6%)	51 (20.32%)	
	III	102 (20.36%)	57 (22.8%)	45 (17.93%)	
	IV	271 (54.09%)	132 (52.8%)	139 (55.38%)	
	Unknown	14 (2.79%)	5 (2%)	9 (3.59%)	

### 2.2 Acquisition of cuproptosis‐related lncRNAs

Nineteen genes associated with cuproptosis have been reported in the literature.^20^, ^21^, ^22^ We collected these genes and labeled them as cuproptosis‐related genes. The expression levels of these genes and lncRNAs were investigated to assess the cuproptosis‐related lncRNAs following co‐expression analysis. Limma package was used for analysis (|correlation coefficients| > 0.4 and *p* < 0.001), and the cuproptosis‐related lncRNAs that could be used for prognosis were detected using the univariate Cox regression analysis method (threshold: *p* < 0.05).

### 2.3 Development of the risk model and construction of the prognostic signature

Data overfitting for the case of the training cohort was eliminated using the Least Absolute Shrinkage and Selection Operator (LASSO) regression. The multivariate Cox analysis algorithm was used to streamline the amount and calculate the coefficients corresponding to the cuproptosis‐related lncRNAs. Six prognostic cuproptosis‐related lncRNAs (AL109936.2, CDKN2A‐DT, AC090587.1, KLF3‐AS1, AL133395.1, and LINC01063) were selected for the construction of the risk model. The coefficients were determined using the Cox proportional hazard regression analysis algorithm, and the coefficients were used for calculating the risk score using the following formula:

$$\;\text{Risk score}=\sum_{i=1}^{n}\text{coefi}\times \text{cuproptosis}-\text{related lncRNA expression}.$$

### 2.4 Comprehensive analysis of the total cohort

The total cohort was used for further evaluation. The performance of the risk model in determining the risk status was studied using the principal component analysis (PCA). Heatmaps were analyzed to determine the lncRNA levels in the risk groups. The recorded Kaplan–Meier curves were analyzed, and the log‐rank test was conducted to compare the OS, DSS, and PFS values. The prognostic value of the risk model was analyzed using the Cox regression analysis. We used a nomogram to predict the 1‐year, 3‐ year, and 5‐year OS of HNSCC patients. The “survival” and “rms” packages were used to arrive at the results, and the risk score and clinicopathologic features (age, gender, T stage, N stage, lymph node metastasis, and clinical stage) were taken into account during the data analysis process. The calibration plots, time‐dependent receiver operator characteristic (ROC) curves, and C‐index were analyzed to identify the diagnostic accuracy of the nomogram.

### 2.5 Correlation between the risk model and clinicopathologic features

Chi‐square tests were used to determine the correlation between the risk model and clinicopathologic features (gender, age, N and T stages, lymph node metastasis, clinical stage, etc.). The distribution of clinicopathological features in each HNSCC sample was presented using a heatmap. The Kaplan–Meier curves were generated, and log‐rank tests were conducted to compare the OS between the high‐ and low‐risk groups (clinical stage subtype) using the “survival” and “survminer” packages.

### 2.6 Functional and pathway enrichment analysis

Specific criteria were taken into account to screen the differentially expressed genes (DEGs) across the two risk cohorts (criteria: FDR *q* < 0.05; |log2FC| > 1). The “limma” package was used to arrive at the results. Subsequently, Gene Ontology (GO) and Kyoto Encyclopedia of Genes and Genomes (KEGG) enrichment analyses were conducted. The “clusterProfiler” and “ggplot2” packages were used for analysis. The identified DEGs were used to evaluate the risk model and functional pathways associated with HNSCC.

### 2.7 Correlation between the immune cell infiltration and risk model

The contents of 22 immune cell types (corresponding to each of the HNSCC samples belonging to the risk groups) were estimated using the CIBERSORT algorithm.^23^ The single sample gene‐set enrichment analysis (ssGSEA) score was conducted. Subsequently, 13 immune‐related gene sets were compared using “GSVA” to quantify various immune pathways for HNSCC samples.^24^

### 2.8 Correlation between the TMB, chemotherapy, and risk model

The hg19 reference genome was used for data annotation. The “GenVisR” package was used to visualize the data corresponding to somatic mutation. The mutated genes were identified using the “maftool” package, and the top 15 genes characterized by the maximum mutation frequencies were displayed in the waterfall charts. The TMB was determined by dividing the sum of mutations by the exome size (38 megabases).^25^ The “survival” and “survminer” packages were used to evaluate the OS values. The results were influenced by the TMB values and risk scores, and the OS values were determined by analyzing the Kaplan–Meier curves and conducting the log‐rank tests. The half‐maximum inhibitory concentration (IC_50_) values of the four commonly used chemotherapeutics (cisplatin, docetaxel, gemcitabine, and paclitaxel) were determined to ascertain whether the risk model could act as a potential biomarker during the process of drug screening. The “pRRophetic” package^26^ was used to determine the results, and the values obtained for the two risk groups were compared.

### 2.9 Statistical analysis

R (version 4.1.0) was used for data analyses. This software was also used to generate the relevant charts. The Kaplan–Meier survival curves were generated based on the results obtained from the log‐rank test. The test results were also used to evaluate the differences in the OS, DSS, and PFS values. The Chi‐square test or the Wilcoxon signed‐rank test was conducted to distinguish the subgroups. The correlation between the risk score and different clinical factors was studied using Univariate and multivariate Cox regression analyses. The area under the curve (AUC) and the receiver operating characteristic (ROC) curve were analyzed to determine the predictive accuracy of the risk model (statistical significance: *p* < 0.05).

## 3 RESULTS

### 3.1 Identification of the six cuproptosis‐related lncRNAs

**FIGURE 1:**
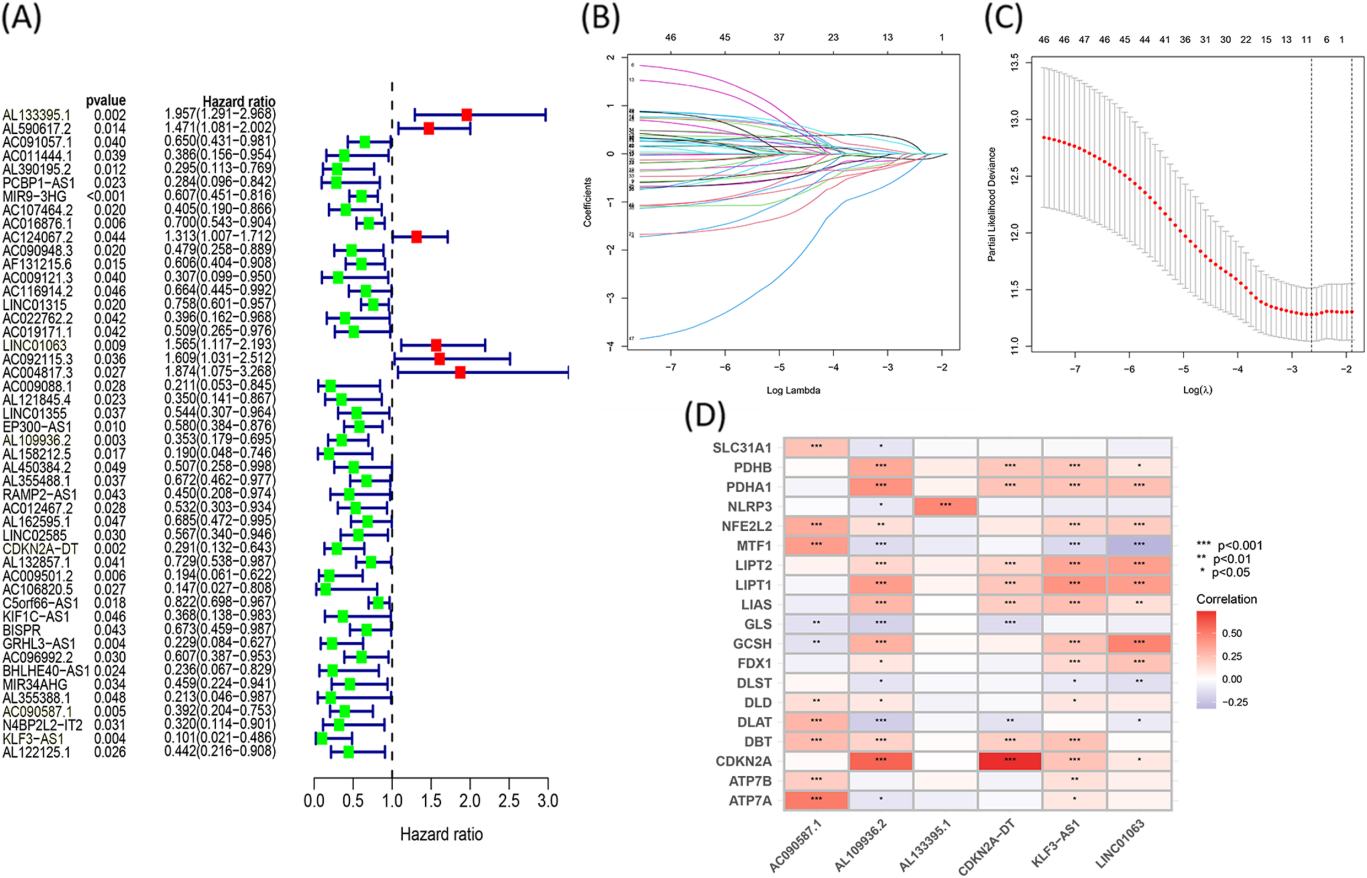
Identification of six cuproptosis‐based lncRNA signature for HNSCC patients. (A) Forest map shows the prognostic value corresponding to the cuproptosis‐related lncRNAs (univariate Cox regression analysis); *p* < 0.05. (B) LASSO Cox regression was conducted by computing the minimal criterion. (C) Generalized cross‐validation curve of paired likelihood deviance; (D) Correlation between the six lncRNAs and cuproptosis‐related genes in the training cohort

The expressions of the previously reported cuproptosis‐related genes (19 in total) were obtained from the TCGA cohort. Following Pearson correlation analysis, a total of 524 lncRNAs were identified to correlated with one or more of the cuproptosis‐related genes. The univariate Cox regression analysis method was followed for further screening, and 48 lncRNAs were identified and labeled as cuproptosis‐related prognostic lncRNAs (*p* < 0.05) (Figure 1A). The HNSCC cohort containing 501 samples was randomly divided into the training and testing cohorts (ratio: 1:1). The LASSO Cox regression analysis method was followed to reduce the variables in the training cohort. This was achieved by introducing the lambda value (Figure 1B,C). Finally, six lncRNAs were identified, and the predictive signature risk model (weighted based on the coefficients determined using the regression analysis method) was developed on the basis of the identified lncRNAs. The correlation of the six lncRNAs in the signature with the cuproptosis‐related genes is shown in Figure 1D.

### 3.2 Analysis of the six cuproptosis‐related lncRNAs

**FIGURE 2:**
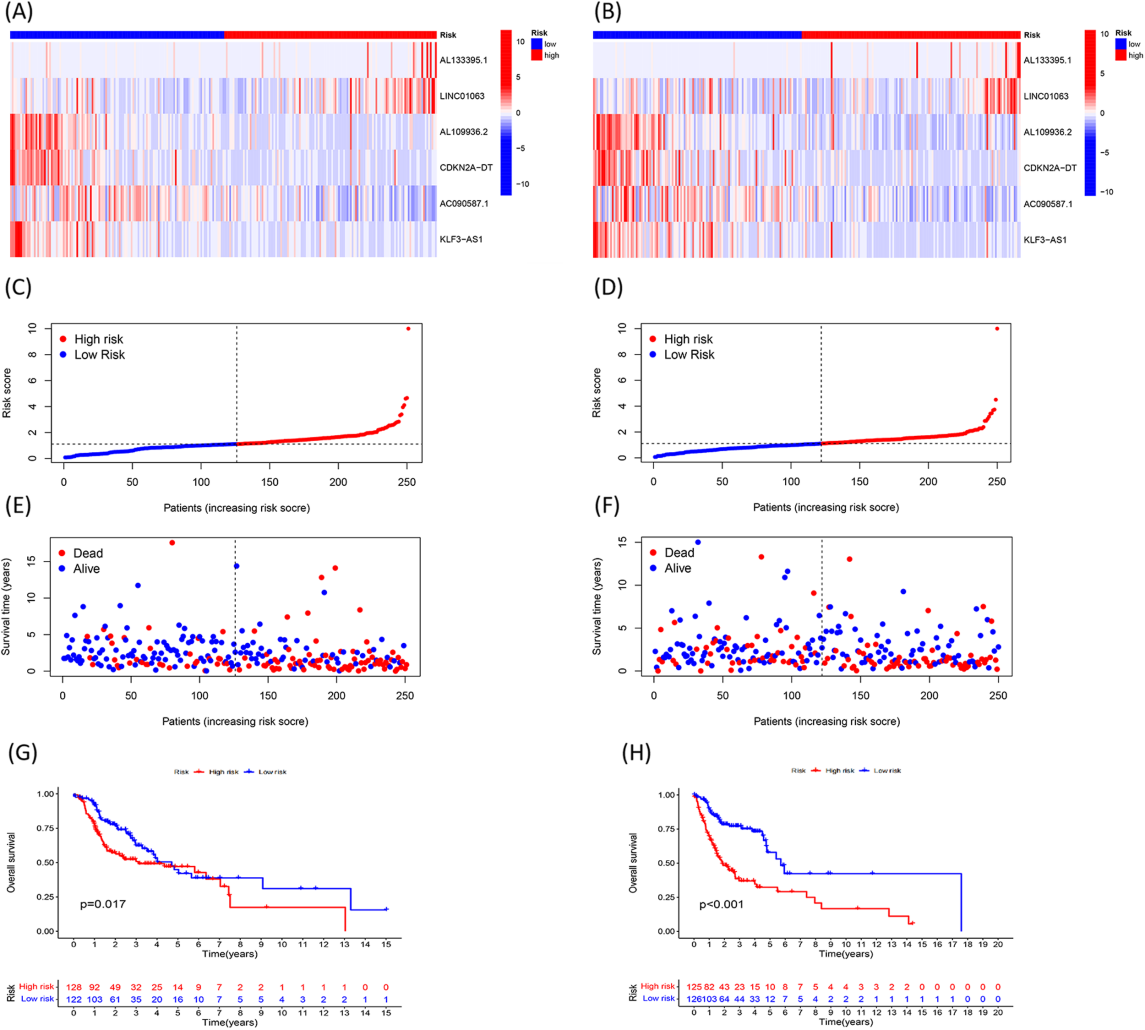
Prognosis data obtained using the six cuproptosis‐related lncRNA‐based signature in the training and testing cohorts. Heatmap shows the expression comparison of the six lncRNAs in the high‐ and low‐risk groups in the training (A) and testing cohorts (B). The distribution and median values of the risk scores in the training (C) and testing cohorts (D); The distributions of overall survival status and overall survival time in the training (E) and testing cohorts (F); Kaplan–Meier curve presents the overall survival of high‐ and low‐risk HNSCC patients in the training (log‐rank, *p* = 0.017) (G) and testing cohorts (log‐rank, *p* < 0.001)(H).

Figure 2A presents the expression levels of the six lncRNAs belonging to the two risk groups in the training cohort, and Figure 2B presents the corresponding data for the testing cohorts. We observed that four lncRNAs (AL109936.2, CDKN2A‐DT, AC090587.1, and KLF3‐AS1) belonging to the low‐risk group were up‐regulated, while two lncRNAs were down‐regulated (AL133395.1 and LINC01063) in the two cohorts. The survival duration of the patients suffering from HNSCC and characterized by a high‐risk score was shorter than the survival time recorded for the patients characterized by low‐risk scores. The death rates recorded for the former were higher than those recorded for the latter. This was true for the patients belonging to both the cohorts (training cohort, Figure 2C,E; testing cohort, Figure 2D,F). Analysis of the Kaplan–Meier curve confirmed that the survival duration recorded for the low‐risk group was longer than the survival duration recorded for the other group for the case of the testing (log‐rank, *p* < 0.001, Figure 2H) and training (log‐rank, *p* = 0.017, Figure 2G) cohorts.

### 3.3 Independent prognostic value of the identified lncRNAs for the total cohort

**FIGURE 3:**
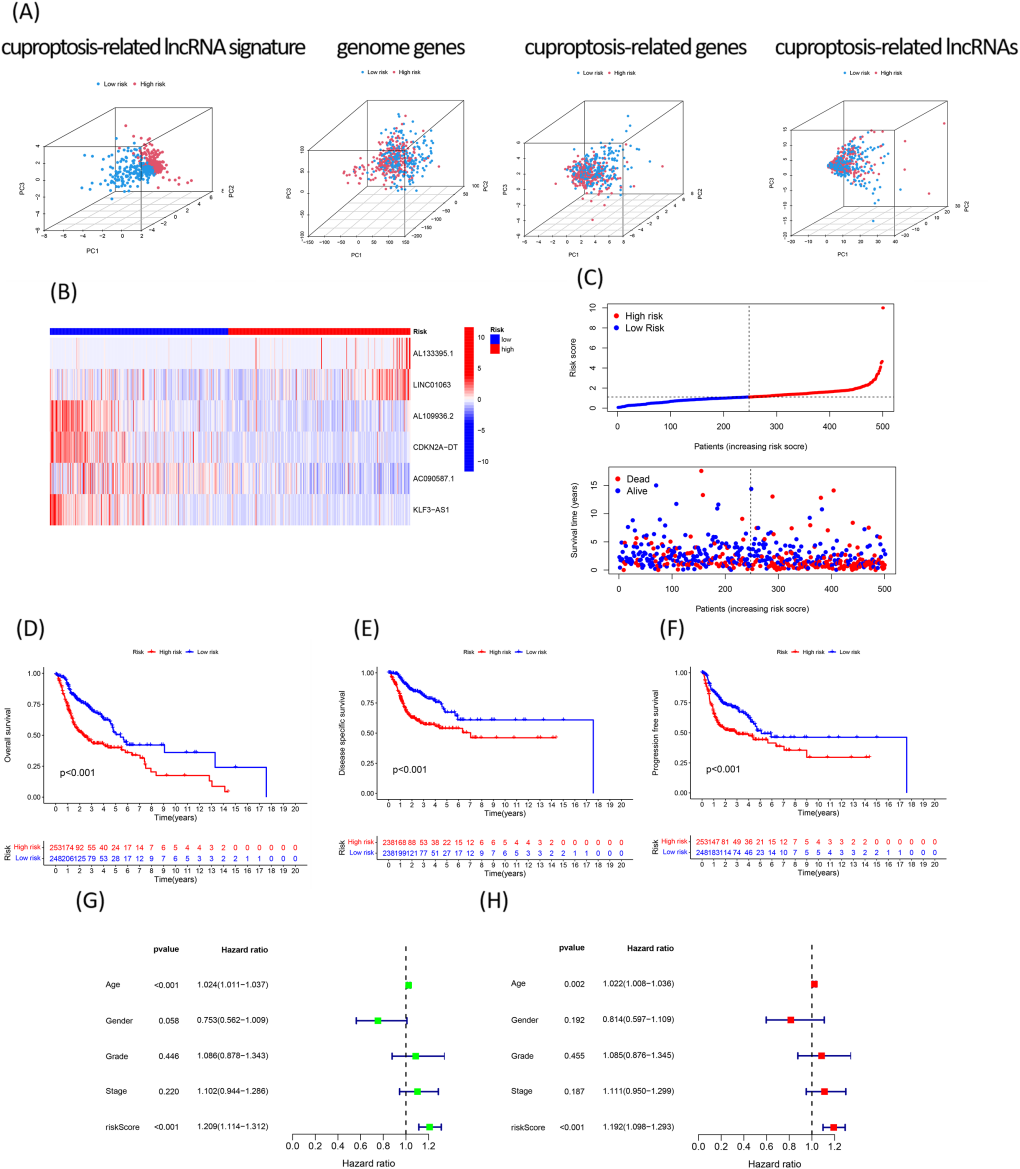
Independent prognostic values corresponding to the six cuproptosis‐based lncRNA signature for the HNSCC patients in the total cohort. (A) PCA analyses indicated that the six cuproptosis‐related lncRNAbased signature could be better distinguished by the risk status of the patients than the whole genome expression, cuproptosis‐related genes, and cuproptosis‐related lncRNAs. (B) Heatmap displays the expressions of the six lncRNAs in the high‐ and low‐risk groups. (C) Distributions of risk score, overall survival status, and overall survival time in the total cohort. Kaplan–Meier curves show the overall survival (D); disease‐specific survival (E), and progression‐free survival (F) (log‐rank, *p* < 0.001). (G) Univariate Cox regression analysis of the cuproptosis‐related lncRNA signature for all samples; (H) Multivariate Cox regression analysis of the cuproptosis‐related lncRNA signature

A rough estimate of the prognosis value of the signature was obtained. Following this, the samples in the two cohorts were integrated to form the total cohort, and this cohort was used for subsequent studies. The PCA method was used to determine whether the constructed signature could be used to determine the risk status efficiently. It was inferred that the risk status of the patients could be efficiently determined using the signature. However, the differences in the risk status values could not be efficiently determined by analyzing the whole genome expression, cuproptosis‐related genes, and cuproptosis‐related lncRNAs (Figure 3A). Four lncRNAs were up‐regulated, and two lncRNAs were down‐regulated (Figure 3B) in the total cohort, and these results were consistent with the results previously reported in this research.

A scatter plot was generated, and the analysis of the plot revealed that the accuracy of prognosis recorded for the low‐risk samples was better than the accuracy of prognosis recorded for the high‐risk group. The mortality rates of the high‐risk group patients were higher than the mortality rates of the low‐risk group patients in the total cohort (Figure 3C). It was observed that the patients characterized by low‐risk scores exhibited longer OS times (log‐rank, *p* < 0.001, Figure 3D), DSS times (log‐rank, *p* < 0.001, Figure 3E), and PFS times (log‐rank, *p* < 0.001, Figure 3F) than the patients characterized by high‐risk scores. Results indicated that the signature was characterized by an independent prognostic value for the case of the total HNSCC cohort (univariate HR = 1.209, Figure 3G; multivariate HR = 1.192, Figure 3H; *p* < 0.001).

### 3.4 Nomogram‐combined risk score and clinical characteristics for predicting the survival and prognostic accuracy of HNSCC patients in the total cohort

**FIGURE 4:**
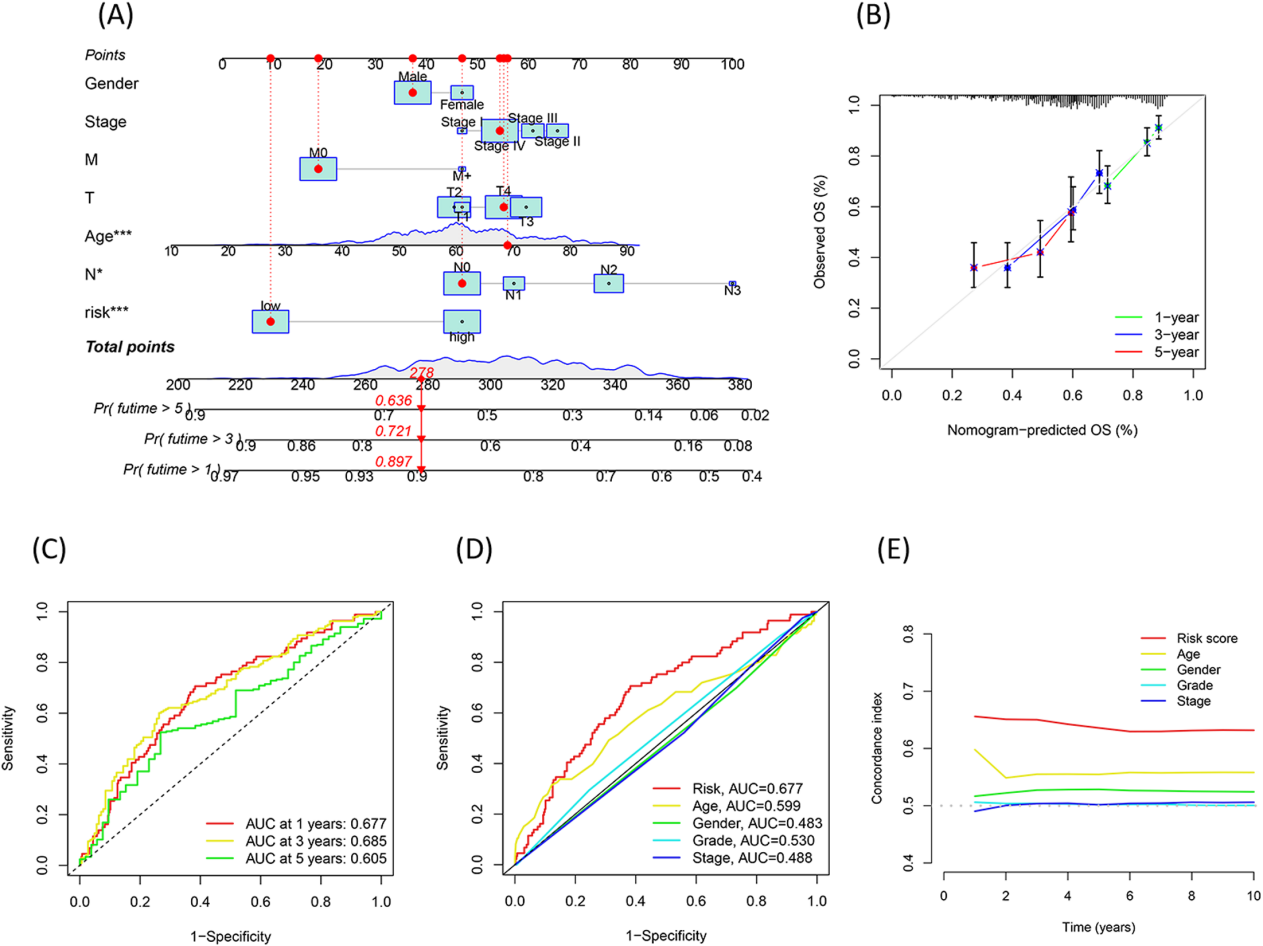
Nomogram‐combined risk score and clinical characteristics for predicting the survival and prognostic accuracy of the HNSCC patients in the total cohort. (A) Nomogram‐combined risk score and clinical information; (B) Calibration curves corresponding to the nomogram for predicting the 1‐, 3‐, and 5‐ year overall survival; (C) AUC of time‐dependent ROC curves for the survival prediction of the risk score; (D) AUC of ROC curves compared the prognostic accuracy of the risk score and clinical features. (E) C‐index compared the prognostic accuracy of the risk score and clinical factors.

Risk scores and clinical factors (such as age, gender, T stage, N stage, and lymph node metastasis) were taken into account during the construction of a nomogram for predicting the survival rates of patients with HNSCC (Figure 4A). Analysis of the calibration chart (Figure 4B) indicated that the predicted curve was next to the 45‐degree line (ideal curve). This indicated that the nomogram agreed well with the ideal model. Analysis of the time‐dependent ROC curves revealed the AUC values (0.677, 1 year; 0.685, 3 years; 0.605, 5 years) (Figure 4C). The values indicated that the nomogram yielded highly accurate results. Furthermore, analysis of the ROC curves and other clinical factors confirmed the excellent prediction performance of the risk score (Figure 4D,E, age [0.599], gender [0.483], grade [0.530], and stage [0.488]).

### 3.5 Correlation between the risk score and clinicopathologic features associated with HNSCC

**FIGURE 5:**
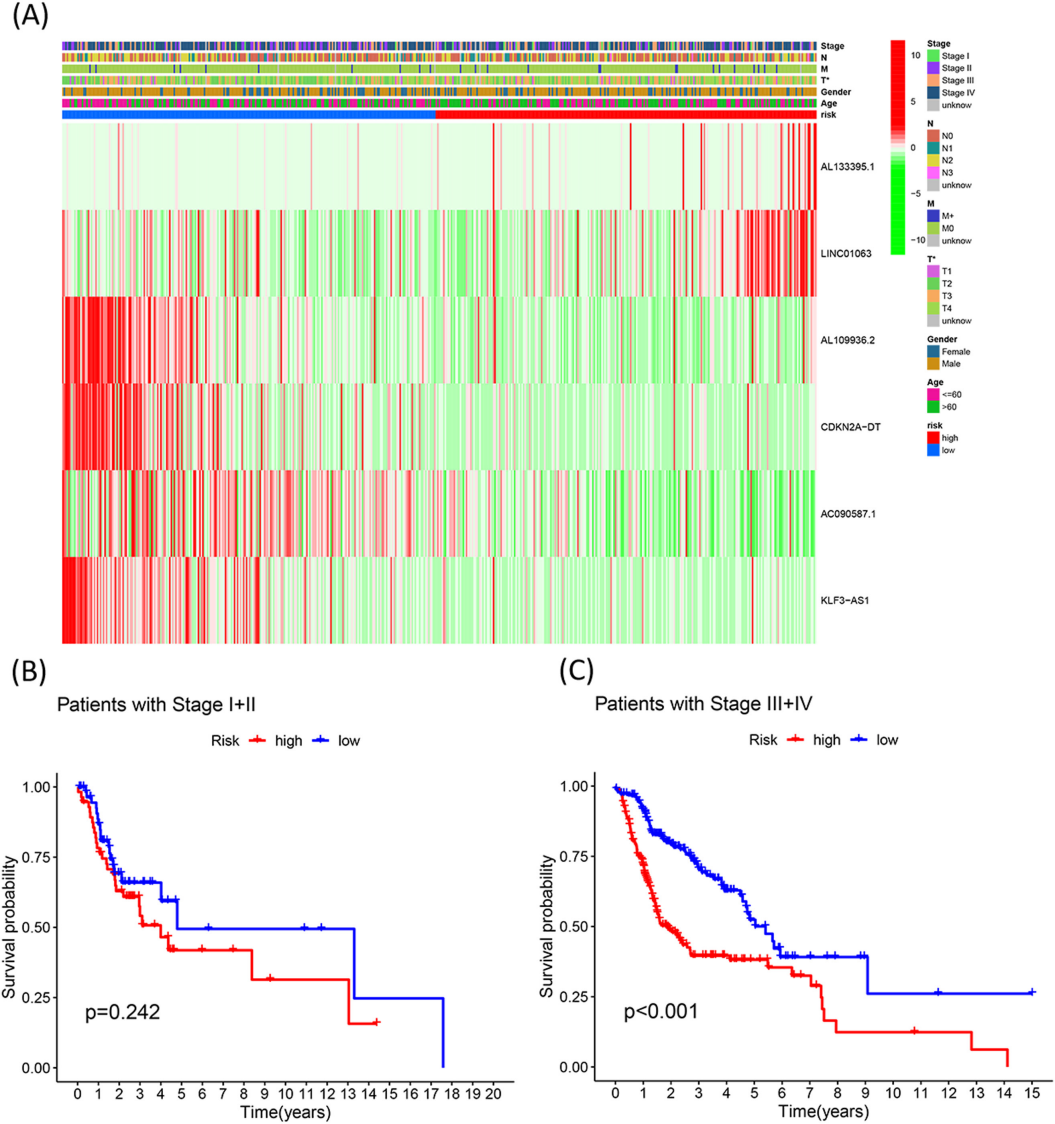
Correlation between the risk score and clinicopathologic features of HNSCC. (A) Heatmap presents the link between the six cuproptosis‐related lncRNAs and clinicopathologic factors corresponding to the HNSCC patients belonging to the high‐ and low‐risk groups. Kaplan–Meier curves corresponding to clinical subtypes for stages I + II (log‐rank, *p* = 0.242) (B) and III + IV (log‐rank, *p* < 0.001) (C).

To study the clinical application of the cuproptosis‐related lncRNA signature, the correlation between the risk score and clinical factors was analyzed and presented in a heatmap in Figure 5A. Analysis of the Kaplan–Meier curve helped compare the OS of the patients in the two risk groups. The results revealed that the prognosis for the low‐risk score samples was better than the prognosis for the high‐risk score samples belonging to the stage III + IV subgroups (log‐rank, *p* < 0.001, Figure 5C). However, a significant difference between the stage I + II subgroups was not observed (*p* = 0.242, Figure 5B).

### 3.6 Functional enrichment of cuproptosis‐related lncRNA signature

**FIGURE 6:**
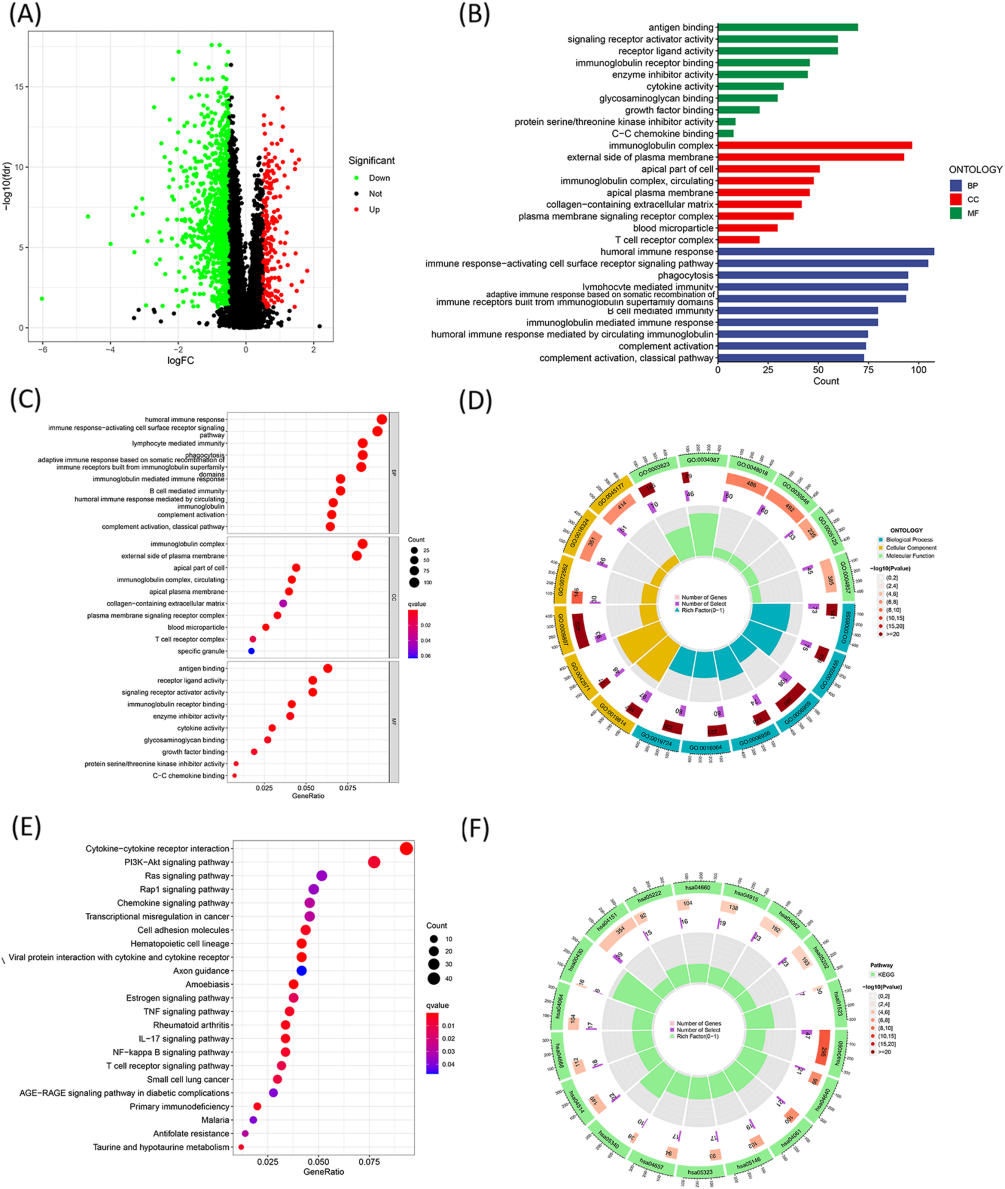
Functional enrichment of cuproptosis‐related lncRNA signature. (A) Volcano plot corresponding to the differentially expressed genes between high‐ and low‐risk groups. (B) GO enrichment of differentially expressed genes in biological process (BP), cellular component (CC), and molecular function (MF). Bubble chart (C) and GO circle plot (D) of differentially expressed genes between the two groups obtained from GO analysis. Bubble chart (E) and KEGG circle plot (F) corresponding to differentially expressed genes between the two groups obtained following the KEGG analysis.

Differentially expressed genes (1379 in total) were identified between the high and low‐risk groups (Figure 6A). The GO analysis method was used to determine the distribution of the DEGs in functional enrichment level (biological process (BP), cellular component (CC), and molecular function (MF); Figure 6B). Enrichment of several immune‐related biological processes such as B cell‐mediated immunity, lymphocyte‐mediated immunity, immune response‐activating cell surface receptor signaling pathway, immunoglobulin mediated immune response, and humoral immune response, and adaptive immune response attributable to somatic recombination of immune receptors originating from the immunoglobulin superfamily domains was observed (Figure 6C,D). Results obtained from the KEGG analysis revealed that these DEGs were significantly enriched in the tumor‐associated pathways. The top 23 KEGG terms are presented in Figure 6E,F.

### 3.7 Correlation between the cuproptosis‐related lncRNA signature and immune function

**FIGURE 7:**
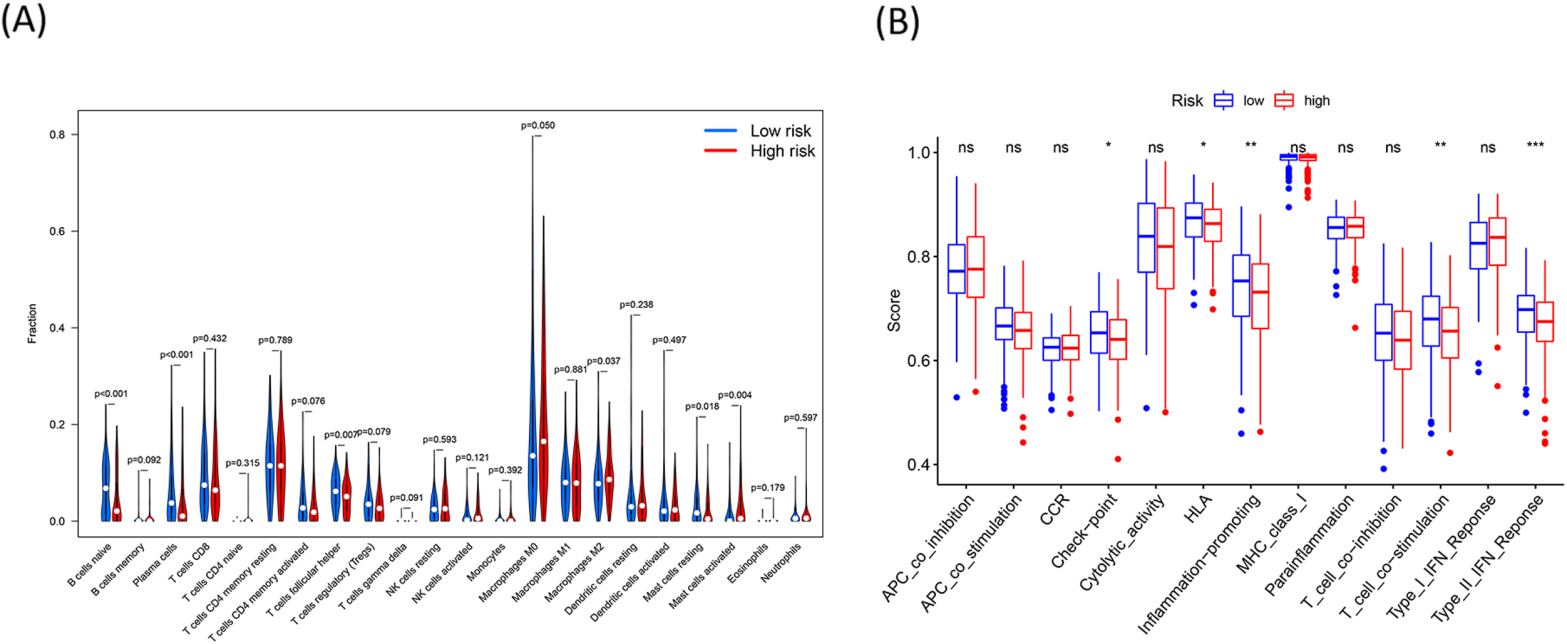
Correlation between the cuproptosis‐related lncRNA signature and immune function. (A) Violin plot shows the 22 infiltrated immune cells associated with the high‐ and low‐risk patients. (B) Box plot shows the risk score corresponding to the 13 immune‐related functions. Adjusted *p* values were showed as: ns, not significant; *p < 0.05; **p < 0.01; ***p < 0.001

The immune cell infiltration levels corresponding to the two risk groups were determined. The violin plot presents the 22 immune cells (Figure 7A). We observed a significant increase in the abundance of naive B cells (*p* < 0.001), follicular helper T cells (*p* = 0.007), and resting mast cells (*p* = 0.018) in the low‐risk patients. Increased plasma cell abundance (*p* < 0.001) was also observed in these patients. It was also observed that under these conditions, the abundance of activated mast cells (*p* = 0.004) and M2 macrophages (*p* = 0.037) decreased significantly. We also observed that the checkpoint, human leukocyte antigen (HLA), T cell co‐stimulation, inflammation‐promoting, and type II interferon response pathways were enriched in the low‐risk group. The results were arrived at using the ssGSEA analysis (Figure 7B).

### 3.8 Relationship between TMB and the risk model

**FIGURE 8:**
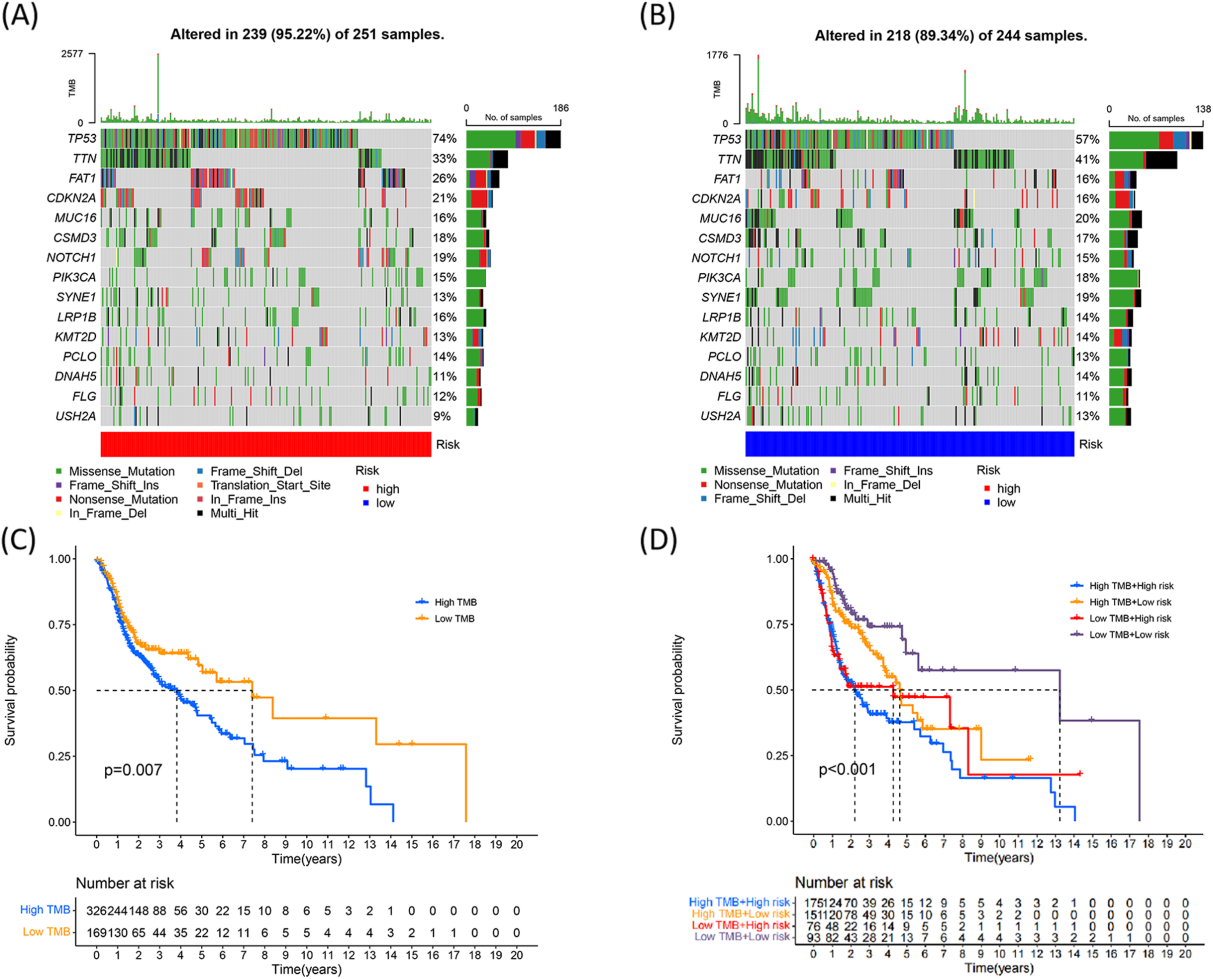
Relationship between the risk model and TMB in HNSCC. Waterfall plot presents the top 15 mutation genes in the high‐risk (A) and low‐risk (B) groups. (C) Kaplan–Meier curves present the overall survival for the high‐ and low‐TMB groups (log‐rank, *p* = 0.007). (D) Overall survival corresponding to HNSCC patients classified by the risk scores and TMB states (log‐rank, *p* < 0.001).

Somatic mutation data were obtained to determine the mutation frequency of the genomic genes corresponding to the risk model. Waterfall plots were generated, and the top 15 mutation genes (Figure 8A and Figure 8B, high‐ and low‐risk groups, respectively) were presented in these plots. A significant difference in the risk groups was not observed. However, it was observed that the mutation frequency corresponding to *TP53* and *TTN* was high in the two groups. The mutation frequency of *TP53* in the high‐risk group was recorded to be 74%. We analyzed the TMB values recorded for each HNSCC patient and divided the patients into high‐ and low‐TMB groups based on the results. Analysis of the Kaplan–Meier curves brought to the forefront that the low‐TMB group patients were more likely to survive for a longer period of time than those in the high‐TMB group (log‐rank, *p* = 0.007, Figure 8C). According to the subgroup survival analysis, the low TMB patients had a better survival time either with high‐ or low‐risk subgroups (log rank, *p* < 0.001, Figure 8D).

### 3.9 Influence of cuproptosis‐related lncRNA signature on chemotherapeutics

**FIGURE 9:**
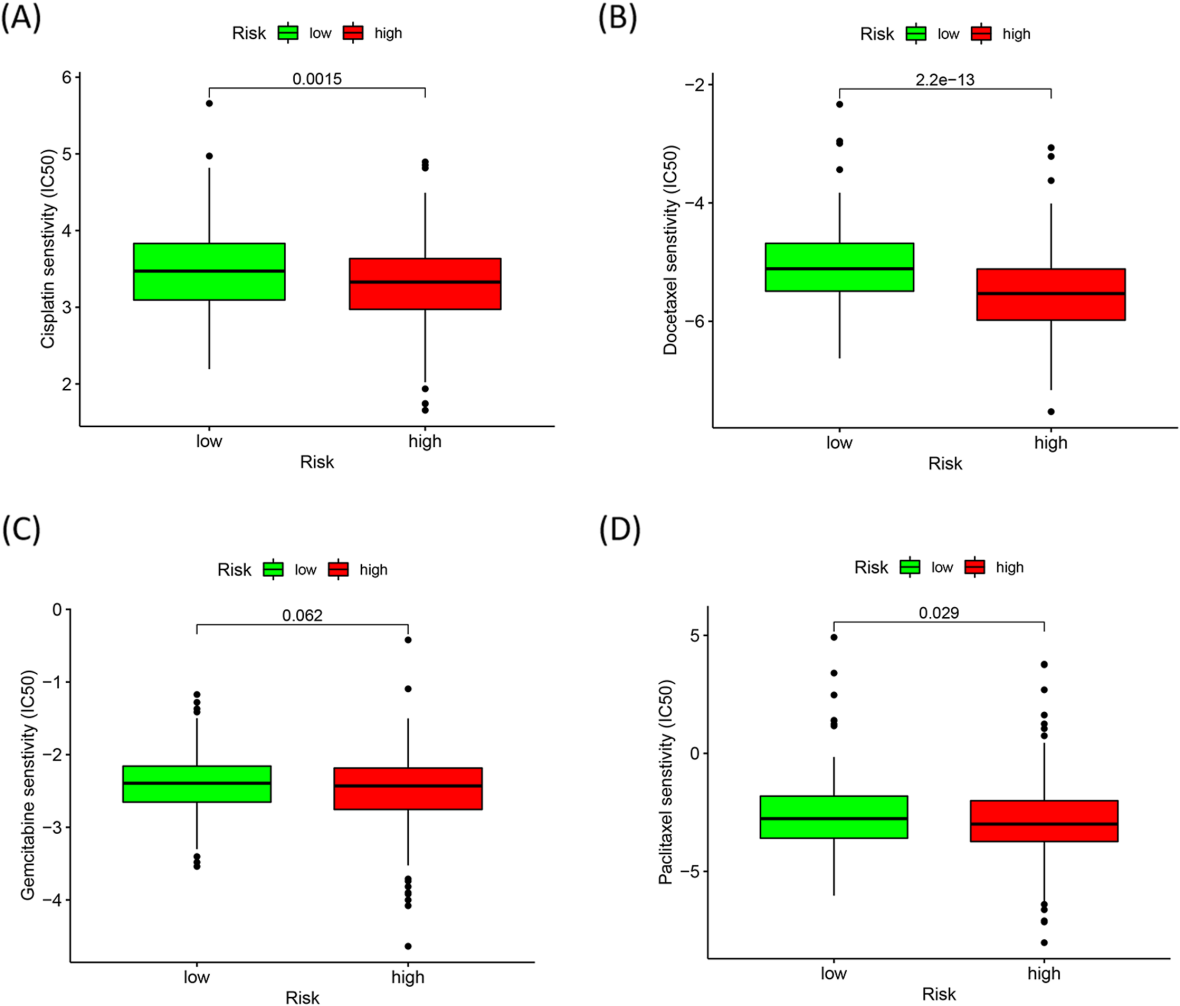
Evaluation of the influence of the cuproptosis‐related lncRNA signature on chemotherapeutics. Drug sensitivity analyses for cisplatin (A), docetaxel (B), gemcitabine (C), and paclitaxel (D) for HNSCC patients belonging to the high‐risk and low‐risk groups.

The IC_50_ values corresponding to four common chemotherapeutics agents (cisplatin, paclitaxel, docetaxel, and gemcitabine) were determined for each patient using the pRRophetic algorithm. As shown in Figure 9, the IC_50_ values for cisplatin (Figure 9A; *p* = 0.0015), docetaxel (Figure 9B; *p* = 2.2e‐13), and paclitaxel (Figure 9D; *p* = 0.029) recorded for the high‐risk group were lower than those recorded for the low‐risk group. This indicated that the high‐risk patients were more sensitive to cisplatin‐, docetaxel‐, and paclitaxel‐based treatment schemes. For gemcitabine (*p* = 0.062; Figure 9C), there was no obvious correlation between the IC_50_ values and the low‐ and high‐risk groups.

## 4 DISCUSSION

Radiotherapy, chemotherapy, targeted therapy, and various immunotherapies have been used over the years to treat patients with HNSCC.^27^ Although significant progress has been made in the field of treating HNSCC, the clinical prognosis is poor. This can be attributed to the high malignancy rate, high metastasis rate, and high heterogeneity in HNSCC patients.^28^ Age, TNM staging, gender, and grade are the commonly used prognostic factors. However, these parameters cannot be used to accurately predict the survival rates as different results are obtained for patients characterized by similar TNM stages.

To the best of our knowledge, researchers have recently started focusing on the relationship between cuproptosis and several cancers, and constructed some different cuproptosis‐related gene signatures for predicting prognosis in cervical cancer,^29^ pancreatic cancer,^30^ Clear Cell Renal Cell Carcinoma.^31^ LncRNAs are potential biomarkers that can be used to treat numerous diseases, especially tumors.^8^, ^9^ We identified 48 cuproptosis‐related lncRNAs that were dysregulated under conditions of HNSCC. Further screening helped us identify six lncRNAs (AL109936.2, CDKN2A‐DT, AC090587.1, KLF3‐AS1, AL133395.1, and LINC01063) that were closely related to the cuproptosis‐related genes. Few researchers have investigated the roles played by AL109936.2 and AL133395.1 in the onset and advancement of different types of cancers. Some researchers have explored the roles of KLF3‐AS1 and LINC01063 in the onset and progression of different types of cancers. KLF3‐AS1 is down‐regulated in gastric cancer, and this gene can hinder the process of tumor progression and improve chemoresistance in gastric cancer.^32^ Mao et al.^33^ reported that KLF3‐AS1 present in the exosomes secreted from human mesenchymal stem cells could help reduce the extent of pyroptosis in cardiomyocytes and improve myocardial infarction by exploiting the miR‐138‐5p/Sirt1 axis. LINC01063 was associated with ferroptosis^34^, ^35^, ^36^ and autophagy^37^, ^38^, ^39^ in various tumors. The results suggested that KLF3‐AS1 and LINC01063 could potentially regulate the process of cell death. We observed that cuproptosis was closely associated with KLF3‐AS1and LINC01063. CDKN2A‐DT and AC090587.1 had been reported could act as cuproptosis‐related biomarkers in HNSCC,^40^, ^41^ which was consistent with our findings. However, compare with the signatures with eight or more cuproptosis‐related lncRNAs, our algorithms with six cuproptosis‐related lncRNAs were simpler and easier for verification in the future applications.

We identified an innovative signature based on six differentially expressed lncRNAs associated with cuproptosis in HNSCC samples. The required data were obtained by accessing the TCGA database. The patients were divided into two risk groups based on the median risk score. PCA analysis had proved that the patients could be better distinguished when using the cuproptosis‐related lncRNA signature instead of the whole genome expression, cuproptosis‐related genes, and cuproptosis‐related lncRNAs. Analysis of the Kaplan–Meier curves indicated that the OS, PFS, and DSS values corresponding to the patients in the high‐risk group were shorter than the low‐risk group. The univariate and multivariate Cox regression analyses also confirmed that risk score was an independent prognostic factor in HNSCC patients both in the train, test, and total cohorts. Furthermore, we constructed a nomogram by combining the risk score and clinicopathological factors to analyze the accuracy and applicability of the prediction. The AUC values and C‐index values obtained using the risk model was higher than the values obtained using the clinical factors (age, gender, grade, and clinical stage). This indicated the accuracy of the prediction realized for the prognosis of HNSCC patients. The predictions made using the developed signature were more accurate than the predictions made using clinical markers.

We used the GO and KEGG enrichment analysis to study the mechanisms associated with the risk model. The results indicated that the DEGs were primarily enriched in the immune response and tumor‐associated pathways. We simultaneously explored the relationship between the immune cell infiltration levels and risk scores. The low‐risk group had a higher infiltration level of naive B cells, plasma cells, follicular helper T cells, and resting mast cells. The checkpoint, HLA, inflammation‐promoting, T cell co‐stimulation, and type II interferon response pathways were enriched in the low‐risk group, and the results were arrived at following the ssGSEA analysis method. These results indicated that the patients characterized by low‐risk scores exhibited stronger immune activation ability to trigger anticancer responses than the patients characterized by high‐risk scores. We hypothesized that this could potentially explain the favorable prognosis obtained for the low‐risk group.

TMB affects immune cell infiltration levels, and it can potentially affect clinical responses to immunotherapy.^42^, ^43^ Results revealed that the TMB values for high‐risk score patients were higher than the TMB values corresponding to low risk score patients. TMB has been proposed as a predictive biomarker to identify the response toward immune checkpoint blockade, and this can be attributed to the fact that TMB influences the process of tumor mutations, resulting in the generation of immunogenic neoantigens.^44^ Zhang et al.^45^ reported that the prognosis for high TMB patients was poorer than the prognosis of low TMB patients. This can be attributed to the fact that the processes of B cell and CD4 + T cell infiltration are stimulated by the TMB levels. We also observed that the rate of survival of HNSCC patients categorized under the low‐TMB group was higher than the rate of survival of the patients categorized under the high TMB group. Subgroup survival analysis revealed that the prognosis of high TMB value patients was poorer than the prognosis of low TMB value patients. This was true for both the subgroups.

Chemotherapy is an indispensable component of the HNSCC treatment process, and it has benefited advanced‐stage cancer patients.^46^ The platinum‐based doublet therapy adopted using fluorouracil or taxane is the first‐line chemotherapeutic regimen to which HNSCC patients are subjected.^47^ Evidence suggested that copper and its transporters may affect chemotherapy outcomes.^48^ As a good prognostic predictor, it is better to have a sensitivity efficacy of chemotherapeutic agents in screening patients. The results obtained by analyzing the relationship between the risk model and the four commonly used chemotherapeutic agents indicated that the high‐risk group was more sensitive to cisplatin, docetaxel, and paclitaxel than the low‐risk group. We believe that the developed signature can be efficiently used to predict chemosensitivity. It can potentially promote the development of chemotherapeutic methods for the treatment of HNSCC.

However, there are some limitations to this study. More reliable results could be obtained if an additional validation set was used to confirm the prognostic value. In vitro and in vivo experiments should be conducted to better understand the mechanisms. We plan to address these limitations in the future.

In conclusion, we constructed a novel cuproptosis‐related lncRNAs signature that could act as an independent predictor for patients suffering from HNSCC. The signature can be potentially used to predict chemosensitivity in individual HNSCC patients. It can also help develop individualized treatment methods for HNSCC patients.

## AUTHOR CONTRIBUTIONS

Qi Huang and Zhenhua Wu conceived and designed this study. Qi Huang and Zhenfei Xiang drafted the manuscript. Quanjie You and Ning Zhu analyzed and expressed the data. Kaiyuan Wu, Jianjuan Ren, and Yihua Gui contributed to data analysis. All the authors reviewed and approved the final version of the manuscript.

## FUNDING INFORMATION

Ningbo Medical and Health Brand Discipline (No. PPXK2018‐02), the Zhejiang Province Medical and Health Research Project (No. 2021KY309 and No. 2022KY295), and the Ningbo Natural Science Foundation (No. 2021 J289).

## Data Availability

The data used for our analysis in this study are openly available at public database (https://portal.gdc.cancer.gov/).
